# Serum kidney injury molecule 1 and β_2_-microglobulin perform as well as larger biomarker panels for prediction of rapid decline in renal function in type 2 diabetes

**DOI:** 10.1007/s00125-018-4741-9

**Published:** 2018-10-05

**Authors:** Marco Colombo, Helen C. Looker, Bassam Farran, Sibylle Hess, Leif Groop, Colin N. A. Palmer, Mary Julia Brosnan, R. Neil Dalton, Max Wong, Charles Turner, Emma Ahlqvist, David Dunger, Felix Agakov, Paul Durrington, Shona Livingstone, John Betteridge, Paul M. McKeigue, Helen M. Colhoun, on behalf of the SUMMIT Investigators

**Affiliations:** 1 Usher Institute of Population Health Sciences and Informatics, University of Edinburgh, Edinburgh, UK; 2 Population Health Sciences, University of Dundee, Dundee, UK; 3 MRC Institute of Genetics and Molecular Medicine, University of Edinburgh, Western General Hospital, Crewe Road South, Edinburgh, EH4 2XU UK; 4 Sanofi-Aventis Deutschland GmbH, Frankfurt, Germany; 5 Lund University Diabetes Centre, Lund University, Malmö, Sweden; 6 Ninewells Hospital and Medical School, University of Dundee, Dundee, UK; 7 Pfizer Inc, Cambridge, MA USA; 8 Evelina London Children’s Hospital, Guy’s & St Thomas NHS Foundation Trust, London, UK; 9 Department of Paediatrics, Wellcome Trust-MRC Institute of Metabolic Science, University of Cambridge, Cambridge, UK; 10 Pharmatics Limited, Edinburgh, UK; 11 Division of Cardiovascular Sciences, University of Manchester, Manchester, UK; 12 Department of Endocrinology and Diabetes, University College London Hospitals, London, UK; 13 NHS Fife, Fife, UK

**Keywords:** Clinical science, Epidemiology, Nephropathy, Proteomics/metabolomics

## Abstract

**Aims/hypothesis:**

As part of the Surrogate Markers for Micro- and Macrovascular Hard Endpoints for Innovative Diabetes Tools (SUMMIT) programme we previously reported that large panels of biomarkers derived from three analytical platforms maximised prediction of progression of renal decline in type 2 diabetes. Here, we hypothesised that smaller (*n* ≤ 5), platform-specific combinations of biomarkers selected from these larger panels might achieve similar prediction performance when tested in three additional type 2 diabetes cohorts.

**Methods:**

We used 657 serum samples, held under differing storage conditions, from the Scania Diabetes Registry (SDR) and Genetics of Diabetes Audit and Research Tayside (GoDARTS), and a further 183 nested case–control sample set from the Collaborative Atorvastatin in Diabetes Study (CARDS). We analysed 42 biomarkers measured on the SDR and GoDARTS samples by a variety of methods including standard ELISA, multiplexed ELISA (Luminex) and mass spectrometry. The subset of 21 Luminex biomarkers was also measured on the CARDS samples. We used the event definition of loss of >20% of baseline eGFR during follow-up from a baseline eGFR of 30–75 ml min^−1^ [1.73 m]^−2^. A total of 403 individuals experienced an event during a median follow-up of 7 years. We used discrete-time logistic regression models with tenfold cross-validation to assess association of biomarker panels with loss of kidney function.

**Results:**

Twelve biomarkers showed significant association with eGFR decline adjusted for covariates in one or more of the sample sets when evaluated singly. Kidney injury molecule 1 (KIM-1) and β_2_-microglobulin (B2M) showed the most consistent effects, with standardised odds ratios for progression of at least 1.4 (*p* < 0.0003) in all cohorts. A combination of B2M and KIM-1 added to clinical covariates, including baseline eGFR and albuminuria, modestly improved prediction, increasing the area under the curve in the SDR, Go-DARTS and CARDS by 0.079, 0.073 and 0.239, respectively. Neither the inclusion of additional Luminex biomarkers on top of B2M and KIM-1 nor a sparse mass spectrometry panel, nor the larger multiplatform panels previously identified, consistently improved prediction further across all validation sets.

**Conclusions/interpretation:**

Serum KIM-1 and B2M independently improve prediction of renal decline from an eGFR of 30–75 ml min^−1^ [1.73 m]^−2^ in type 2 diabetes beyond clinical factors and prior eGFR and are robust to varying sample storage conditions. Larger panels of biomarkers did not improve prediction beyond these two biomarkers.

**Electronic supplementary material:**

The online version of this article (10.1007/s00125-018-4741-9) contains peer-reviewed but unedited supplementary material, which is available to authorised users.

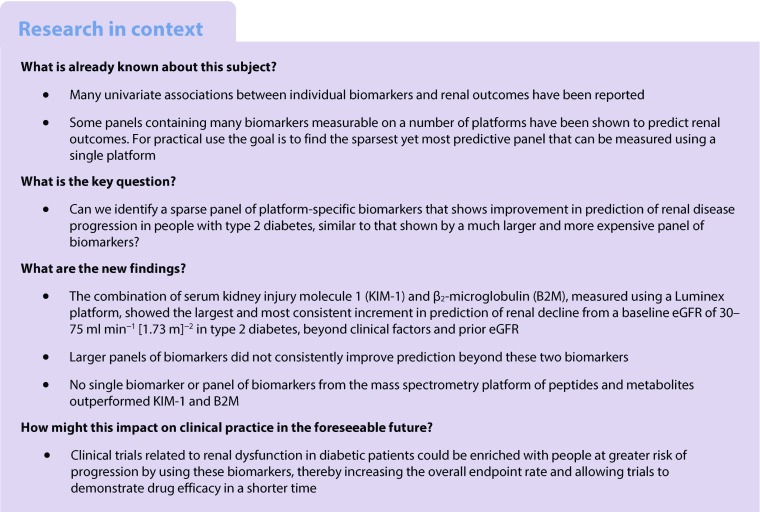



## Introduction

Development of biomarkers predictive of renal disease progression in diabetes would enable enrichment of clinical trials with individuals most at risk [1]. However, the majority of renal biomarker studies have focused on a single biomarker at a time rather than evaluating the potential of large sets of candidates or high-dimensional arrays such as metabolomics panels [2–5].

As part of the Surrogate Markers for Micro- and Macrovascular Hard Endpoints for Innovative Diabetes Tools (SUMMIT) programme http://www.imi-summit.eu/, we previously undertook a nested case–control study in people with type 2 diabetes and chronic kidney disease (CKD; stage 3) at baseline. Therein we identified, from across 207 biomarkers measured by several platforms, which subset maximised prediction of progression in renal function decline on top of both a sparse and an extensive set of clinical covariates [6]. Using forward selection and least absolute shrinkage and selection operator (LASSO) penalised regression approaches, we identified biomarker panels that maximised prediction. Altogether, 42 biomarkers were contained in the two panels we identified using these two approaches.

Since smaller sets of biomarkers that only require a single platform or assay method would be cheaper and more logistically feasible to implement, it is important to consider the extent to which such sparser sets can yield similar gains in prediction to those achieved by large panels found by maximising predictive performance. It is also important to assess whether biomarkers are robust to different biosampling storage conditions, as in our original study all samples had been stored at −80°C. Accordingly, starting with the 42 biomarkers from the previously selected panels we first generated sparse panels of the top five biomarkers from each of the mass spectrometry and then the ELISA/Luminex platforms, respectively, in the original nested case–control study data. Next, we tested the hypothesis that these smaller (*n* ≤ 5), platform-specific combinations might achieve prediction performance similar to that of the larger panels. Specifically, we assessed the performance of these smaller panels, and their subsets, for predicting renal disease progression in three new sample sets that were collected under different sampling and storage conditions and from type 2 diabetes cohorts with different clinical characteristics.

## Methods

### Study populations

The original study was a case–control design nested in the Genetics of Diabetes Audit and Research in Tayside (GoDARTS) cohort, a hospital clinic- and primary care-based cohort of people with diabetes in the Tayside region of Scotland [7]. Here, we also used samples from individuals in GoDARTS who had not been included in the original case–control study and also used an independent set of samples from the Swedish Scania Diabetes Registry (SDR) cohort [8]. For both cohorts, biosamples were collected at the time of study enrolment and were stored according to study-specific protocols [7, 8]. In addition, we used samples from a clinical trial of atorvastatin in people with type 2 diabetes whose eGFR had been measured during follow-up (the Collaborative Atorvastatin in Diabetes Study [CARDS], ClinicalTrial.gov registration no. NCT00327418) [9].

### Phenotype

In the original study [6] we evaluated the performance of biomarkers to predict rapid progression of eGFR defined as ≥40% loss of baseline eGFR within 3.5 years with entrants having a baseline eGFR (calculated by the MDRD4 equation) [10] of 30–60 ml min^−1^ [1.73 m]^−2^ (i.e. CKD3) [6]. Here, we broadened the inclusion criteria to include people with less renal dysfunction at baseline (eGFR 30–75 ml min^−1^ [1.73 m]^−2^) since this range of eGFR is often used for trial entry criteria. Among all participants with this baseline eGFR in these cohorts we compared the ability of biomarkers to predict being a progressor—defined as at least two measures of an eGFR with a > 20% drop from baseline sustained for at least 1 month at any time during follow-up, but within 6 months of each other. Thus, compared with the original study, we are evaluating the biomarkers’ ability to predict a more subtle decline in renal function.

In CARDS, entrants also had a baseline eGFR of 30–75 ml min^−1^ [1.73 m]^−2^, with cases also having had a loss >20% of baseline eGFR during follow-up. However, rather than including all non-cases as for SDR and GoDARTS, control participants were randomly selected from individuals who did not lose >20% of baseline eGFR matched to cases based on baseline eGFR (strata 30–60 and 60–75 ml min^−1^ [1.73 m]^−2^), age (5 year bands) and sex. We used this nested case–control design in CARDS as we had insufficient funds to measure all CARDS samples.

### Clinical covariates

Clinical covariates from the time of sampling were taken from the study-specific databases. HbA_1c_ and serum creatinine were measured as part of clinical care using standard methods. Albuminuria was assessed by either a urinary albumin concentration on a spot urine or a 24 h urinary protein concentration with albuminuria status based on the highest level of albuminuria (normo-, micro- or macroalbuminuria) recorded in the 5 years prior to baseline. Smoking status was based on self-report. Medication data was available from the GoDARTS cohort based on primary care prescribing data and from the CARDS study from self-report at enrolment.

### Laboratory measurement of biomarkers

We measured a total of 42 biomarkers and biomarker ratios that had been included in the large panels generated from the initial SUMMIT study. ELISAs were used for high-sensitivity troponin T using the Roche assay at the University Heart Center Hamburg biomarker laboratory. Multiplexed ELISAs using Luminex technology were used to perform multiplexed, microsphere-based assays for 20 biomarkers as described [11] at the CLIA certified Myriad RBM laboratory (Austin, TX, USA) (see electronic supplementary material [ESM] Methods). Liquid chromatography (LC) electrospray tandem mass spectrometry (MSMS) platforms for targeted metabolite and tryptic peptide analyses were used to measure the remaining 20 biomarkers at the WellChild Laboratory (Kings College London, UK). The ratio of asymmetric dimethylarginine (ADMA) to symmetric dimethylarginine (SDMA) was determined. For GoDARTS and SDR samples all biomarkers were available but for the CARDS samples for budgetary reasons we only measured the Luminex platform biomarkers. Details of the biomarkers and their distribution in the study samples are shown in ESM Table 1. For further details of methods and sample quality control data for the biomarkers measured, see ESM Methods.

### Biomarker data cleaning and imputation

The data from the biomarker laboratories were cleaned and imputed using a sparse iterative regression model before analysis. The iterative imputation model was run ten times, with initial values of the missing at random entries set by sampling from the marginal distribution of the observed values for each variable (see ESM Methods). The dataset used in analysis was the average of the ten imputed sets. All data were Gaussianised prior to analysis by rank transforming each continuous variable and mapping ranks to quantiles of a normal distribution. Generally for almost all biomarkers few samples had undetectable levels or had missing data for other reasons (see ESM Table 1).

### Univariate associations of biomarkers with renal disease progression

We first described univariate associations of the 42 biomarkers being considered with renal disease progression in the three datasets (SDR, GoDARTS and CARDS) separately. Significance was declared at *p* < 0.0012 based on Bonferroni adjustment. Follow-up was partitioned into 1 year time windows with calendar time included in all models as a linear term. We used discrete-time logistic regression models to describe associations examined singly after adjustment for the clinical covariates (age, sex, baseline eGFR, albuminuria and HbA_1c,_ calendar time).

### Generation of a sparse panel from the original case–cohort dataset

Full details of the original case–control study are given elsewhere [6]. Data from that study were used to identify or learn the best performing sparse panels of biomarkers from ELISA- and Luminex-based methods and a panel from the mass spectrometry-based method separately through the same cross-validated forward selection approach used in the original study. We used the R package nestfs (version 0.8.6: https://CRAN.R-project.org/package=nestfs) where the variables are selected based on the smallest false discovery rate computed in an inner cross-validation and stopped the forward selection for each platform at five biomarkers. Then we evaluated performance of the selected panels containing only the first of these five, then the first two, the first three, etc., up to five of these biomarkers in the validation SDR, GoDARTS and CARDS cohorts.

### Predictive performance of sparse biomarker panels in the three validation cohorts

We then evaluated the performance of the sparse panels of biomarkers generated on the original case–control dataset on each of the cohorts. The increment in prediction achieved by the panels was assessed when added to the set of clinical covariates described above and also added to a richer set of clinical covariates (age, sex, baseline eGFR, albuminuria, HbA_1c_, calendar time, diabetes duration, systolic and diastolic blood pressure, BMI, weighted average of historic eGFR, insulin therapy and smoking status). For CARDS samples we included a term for treatment allocation (atorvastatin or placebo) but removed the weighted average of historic eGFR as it was not available. To assess the performance of these biomarker panels in the new datasets, we used tenfold cross-validation to control for overfitting and provide an estimate of predictive performance on data not used to learn the model coefficients. The area under the receiver operating characteristic curve (AUROC) was evaluated by combining risk prediction scores and outcomes in each person-time interval over all test folds. We used the difference in test log-likelihoods to evaluate the strength of evidence favouring one model over another (see ESM Methods). To demonstrate the role of biomarkers in selecting potential clinical trial participants, we plotted the positive predictive value of the test against the percentile of the risk score derived from the logistic regression models with and without biomarkers. As a final comparison step we further considered the performance obtained by the original multiplatform panels on the three cohorts against that for the sparse panels. All analyses were undertaken using R version 3.3.3 (https://www.R-project.org/) [12].

## Results

### Baseline characteristics of the validation cohorts

Clinical characteristics of the participants in the studies are shown in Table 1. Baseline eGFR was similar in the SDR and Go-DARTS cohorts (52.6 vs 53.4 ml min^−1^ [1.73 m]^−2^) and higher in the CARDS participants (62.1 ml min^−1^ [1.73 m]^−2^). The weighted average of prior eGFRs was higher for the Go-DARTS (60.8 ml min^−1^ [1.73 m]^−2^) vs SDR (53.4 ml min^−1^ [1.73 m]^−2^) cohort and albuminuria was slightly more common in the former. Consistent with this, the SDR cohort showed a more rapid loss of renal function than the GoDARTS cohort, with a respective annual decrease in eGFR of 1.3 ml min^−1^ [1.73 m]^−2^ vs 0.5 ml min^−1^ [1.73 m]^−2^. CARDS selected participants with no history of cardiovascular disease (CVD) but at least one CVD risk factor (such as smoking, hypertension or microvascular disease) whereas the other cohorts did not apply these restrictions. Other than differences in baseline characteristics, calendar time of study and country, there are differences in how samples were handled. While GoDARTS and CARDS samples were stored at −80°C, SDR samples were held principally at −20°C. GoDARTS samples were stored for a shorter time than the SDR samples. Accordingly, these cohorts allowed us to test the robustness of any biomarker panel performance across varying conditions. In total there were 403 progression events across the three sample sets—118 in SDR, 192 in GoDARTS and 93 in CARDS.

**Table 1:** Clinical characteristics of the SDR, GoDARTS and CARDS participant sample sets

Characteristic	SDR (*n* = 227)	GoDARTS (*n* = 430)	CARDS (*n* = 183)
Age, years	68.6 (61.6, 75.5)	73.0 (68.0, 78.0)	64.6 (60.8, 69.3)
eGFR, ml min^−1^ [1.73 m]^−2^	52.6 (42.2, 58.5)	53.4 (43.3, 63.8)	62.1 (54.5, 68.7)
HbA_1c_, mmol/mol	61.2 (50.3, 74.9)	54.1 (46.5, 62.6)	58.5 (49.7, 71.6)
HbA_1c_, %	7.7 (6.8, 9.0)	7.1 (6.4, 7.9)	7.5 (6.7, 8.7)
BMI, kg/m^2^	29.0 (26.0, 32.6)	30.6 (27.1, 34.2)	29.0 (26.4, 31.2)
Diabetes duration, years	10.0 (3.0, 15.9)	7.9 (4.5, 13.0)	8.0 (4.0, 11.5)
Systolic blood pressure, mmHg	147.2 (143.7, 151.0)	143.0 (131.1, 155.5)	147.0 (138.0, 160.0)
Diastolic blood pressure, mmHg	79.6 (76.1, 81.5)	71.5 (65.0, 80.0)	82.5 (76.5, 90.0)
Weighted average of historic eGFR, ml min^−1^ [1.73 m]^−2^	53.4 (43.2, 61.3)	60.8 (50.6, 71.8)	NA
Annualised eGFR slope, ml min^−1^ [1.73 m]^−2^ year^−1^	−1.3 (−2.8, 0.1)	−0.5 (−1.9, 0.8)	NA
Year at baseline	1997 (1997, 1998)	2005 (2005, 2007)	1999 (1998, 2000)
Follow-up duration, years	8.7 (4.8, 13.1)	7.1 (5.3, 8.8)	3.2 (2.9, 4.0)
Male sex	83 (36.6)	194 (45.1)	121 (66.1)
Albuminuria, yes	84 (37.0)	133 (30.9)	27 (14.8)
ACEi/ARB use	NA	313 (72.8)	107 (58.5)
Insulin use	88 (38.8)	122 (28.4)	42 (23.0)
Current smoking	10 (4.4)	42 (9.8)	35 (19.1)

Data are median (interquartile range) or *n* (%)

ACEi, ACE inhibitor; ARB, angiotensin II receptor blocker

### Distribution of biomarkers across validation cohorts

ESM Table 1 shows the distribution of the 42 biomarkers included in the analyses, showing that levels of some varied substantially between these cohorts. For adrenomedullin and fibroblast growth factor 23 (FGF23), known to be sensitive to sample handling and storage temperatures, >50% of samples had concentrations below the detection threshold in SDR and CARDS compared with <5% in the GoDARTS cohort. N-terminal prohormone of brain natriuretic peptide (NT-proBNP) also varied with storage temperature across the studies (213 pg/ml, 724 pg/ml and 33 pg/ml for SDR, GoDARTS and CARDS, respectively). There was also a marked difference in the concentrations of glutamine and glutamic acid between the SDR and GoDARTS cohorts, likely reflecting conversion of glutamine to glutamic acid resulting in a higher ratio of glutamic acid to glutamine at higher storage temperatures [13, 14]. However, most other biomarkers, including kidney injury molecule 1 (KIM-1), showed remarkable consistency in range across the three cohorts.

### Univariate associations of biomarkers with eGFR decline

**Fig. 1:**
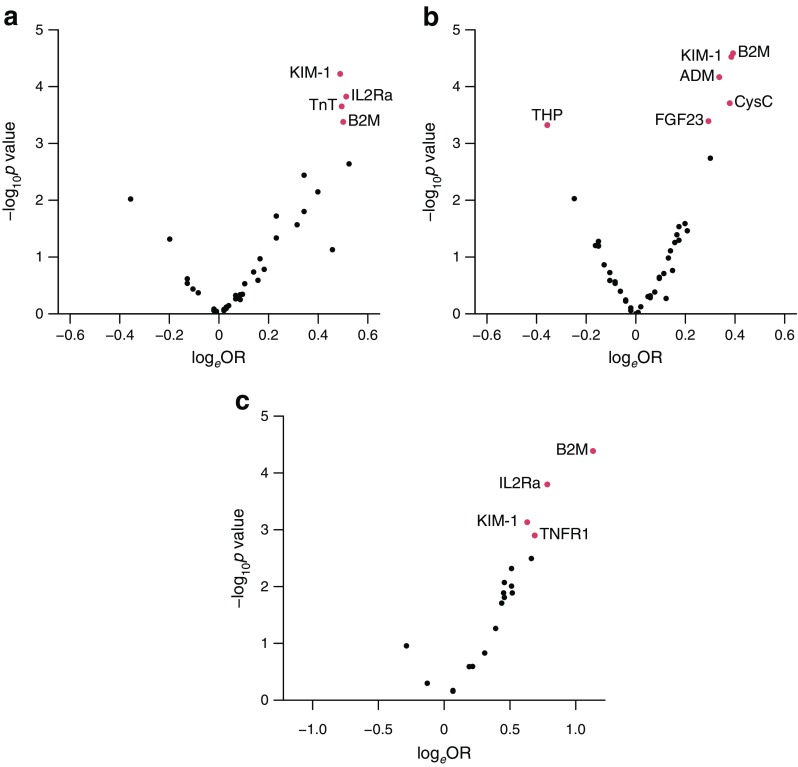
Volcano plots of biomarkers with decline in renal function adjusted for a rich set of clinical covariates in the SDR (**a**), GoDARTS (**b**) and CARDS (**c**) cohorts. The *x*-axes show the OR expressed on a natural logarithm (log_*e*_) scale; the *y*-axes depict the statistical significance on a log_10_ scale. Red circles correspond to biomarkers significantly associated with decline in eGFR (*p* < 0.0012). Clinical covariates for SDR and Go-DARTS cohorts were age, sex, baseline eGFR, albuminuria, HbA_1c_, calendar time, diabetes duration, systolic and diastolic blood pressure, BMI, weighted average of historic eGFR, insulin therapy and smoking status. Clinical covariates for CARDS participants were age, sex, baseline eGFR, albuminuria, HbA_1c_, calendar time, diabetes duration, systolic and diastolic blood pressure, BMI, insulin therapy, smoking status and treatment allocation. ADM, adrenomedullin; CysC, cystatin C; FGF23, fibroblast growth factor 23; THP, Tamm–Horsfall urinary glycoprotein; TNFR1, TNF receptor 1; TnT, high-sensitivity troponin T

Of the 42 biomarkers examined, 12 were significantly associated with decline in eGFR in at least one study, after adjusting for clinical covariates (Table 2). The biomarkers most strongly associated with decline, evaluated singly, were similar across the studies. Of these, beta 2 microglobulin (B2M), cystatin C, IL-2 receptor α (IL2Ra), KIM-1 and Tamm–Horsfall urinary glycoprotein reached the significance threshold in at least two studies. B2M was strongly correlated with eGFR, cystatin C, IL2Ra, TNF receptor 1, adrenomedullin and SDMA. In contrast, KIM-1 and high-sensitivity troponin T were not strongly correlated with any of the other measured biomarkers or clinical covariates. When adjusting for a richer set of clinical covariates, ADMA, SDMA and NT-proBNP were no longer significantly associated with eGFR decline in any of the cohorts (Fig. 1a–c).

**Table 2:** Associations for the 12 biomarkers out of 42 that showed significant univariate association with rapid decline in eGFR

Biomarker	Method	SDR		GoDARTS		CARDS	
		OR (95% CI)	*p* value	OR (95% CI)	*p* value	OR (95% CI)	*p* value
Adrenomedullin (ng/ml)^a^	Luminex	–	–	1.47 (1.26, 1.72)	1.11 × 10^−6^	1.58 (1.09, 2.34)	1.90 × 10^−2^
ADMA (nmol/l)	MSMS	1.46 (1.18, 1.81)	4.60 × 10^−4^	1.04 (0.90, 1.21)	5.97 × 10^−1^	Not measured	–
B2M (μg/ml)	Luminex	1.98 (1.54, 2.56)	1.28 × 10^−7^	1.59 (1.34, 1.89)	1.37 × 10^−7^	2.92 (1.79, 5.06)	4.93 × 10^−5^
Cystatin C (ng/ml)	Luminex	1.83 (1.40, 2.38)	8.08 × 10^−6^	1.64 (1.36, 1.97)	2.03 × 10^−7^	1.84 (1.22, 2.84)	4.31 × 10^−3^
Fibroblast growth factor 23 (ng/ml)	Luminex	1.12 (0.89, 1.41)	3.20 × 10^−1^	1.37 (1.17, 1.59)	7.67 × 10^−5^	1.51 (1.07, 2.15)	2.00 × 10^−2^
IL-2Ra (pg/ml)	Luminex	1.62 (1.28, 2.07)	8.00 × 10^−5^	1.20 (1.02, 1.41)	2.38 × 10^−2^	2.06 (1.42, 3.11)	2.89 × 10^−4^
KIM-1 (ng/ml)	Luminex	1.66 (1.32, 2.09)	1.85 × 10^−5^	1.48 (1.25, 1.76)	6.65 × 10^−6^	1.94 (1.37, 2.81)	2.52 × 10^−4^
NT-ProBNP (pg/ml)	Luminex	1.18 (0.96, 1.45)	1.10 × 10^−1^	1.44 (1.21, 1.71)	3.73 × 10^−5^	1.26 (0.88, 1.82)	2.15 × 10^−1^
SDMA (nmol/l)	MSMS	1.80 (1.33, 2.43)	1.24 × 10^−4^	1.20 (0.98, 1.47)	7.51 × 10^−2^	Not measured	–
Tamm–Horsfall urinary glycoprotein (μg/ml)	Luminex	0.64 (0.50, 0.83)	7.09 × 10^−4^	0.65 (0.53, 0.78)	1.20 × 10^−5^	0.75 (0.54, 1.04)	8.61 × 10^−2^
Troponin T (high sensitivity) (pg/ml)	ELISA	1.66 (1.32, 2.11)	2.33 × 10^−5^	1.05 (0.88, 1.26)	5.63 × 10^−1^	Not measured	–
TNF receptor 1 (pg/ml)	Luminex	1.63 (1.26, 2.13)	2.30 × 10^−4^	1.32 (1.11, 1.56)	1.69 × 10^−3^	1.85 (1.26, 2.82)	2.62 × 10^−3^

Associations after adjusting for clinical covariates (age, sex, baseline eGFR, albuminuria, HbA_1c_, calendar time) are shown; CARDS models also include a term for treatment allocation

^a^For adrenomedullin, >90% samples were below the detection threshold in SDR

MSMS, tandem mass spectrometry

The correlation coefficients for these biomarkers with each other and with baseline eGFR are shown in Table 3.

**Table 3:** Correlation matrix for leading predictive biomarkers for rapid eGFR decline in SDR, GoDARTS and CARDS samples

Biomarker	ADM	ADMA	B2M	CysC	FGF23	FRTN	IL2Ra	KIM-1	MB	NT-proBNP	SDMA	THP	TnT	TNFR1
eGFR														
SDR	–	−0.24	−0.66	−0.66	−0.44	0.10	−0.51	−0.30	−0.55	−0.35	−0.70	0.42	−0.44	−0.64
GoDARTS	−0.49	−0.21	−0.64	−0.67	−0.27	−0.07	−0.38	−0.14	−0.39	−0.33	−0.64	0.41	−0.33	−0.48
CARDS	0.03	–	−0.49	−0.57	−0.05	0.10	−0.37	−0.20	−0.31	−0.13	–	0.12	–	−0.48
ADM^a^														
Go-DARTS	1	0.26	0.70	0.69	0.52	−0.03	0.52	0.21	0.27	0.54	0.48	−0.47	0.42	0.51
CARDS	1	–	0.38	0.05	0.62	0.01	0.25	0.13	−0.09	0.67	–	−0.04	–	0.10
ADMA^b^														
SDR		1	0.35	0.41	0.19	−0.09	0.27	0.07	0.07	0.16	0.48	−0.24	0.19	0.34
Go-DARTS		1	0.31	0.26	0.15	0.04	0.30	0.01	0.06	0.22	0.37	−0.13	0.13	0.18
B2M														
SDR			1	0.89	0.46	0.00	0.62	0.43	0.52	0.43	0.69	−0.49	0.56	0.70
Go-DARTS			1	0.87	0.38	0.05	0.62	0.27	0.34	0.41	0.61	−0.54	0.42	0.65
CARDS			1	0.67	0.36	0.07	0.61	0.25	0.27	0.45	–	−0.27	–	0.57
CysC														
SDR				1	0.48	0.00	0.59	0.42	0.60	0.38	0.71	−0.48	0.53	0.69
Go-DARTS				1	0.37	0.12	0.56	0.25	0.41	0.41	0.60	−0.51	0.42	0.66
CARDS				1	0.15	0.06	0.52	0.18	0.42	0.17	–	−0.21	–	0.46
FGF23														
SDR					1	−0.13	0.28	0.23	0.32	0.37	0.39	−0.29	0.33	0.43
Go-DARTS					1	−0.23	0.29	0.20	0.10	0.37	0.24	−0.23	0.21	0.31
CARDS					1	−0.17	0.24	0.25	−0.01	0.53	–	−0.02	–	0.19
FRTN														
SDR						1	0.01	0.27	0.13	−0.04	0.03	0.03	0.13	−0.04
Go-DARTS						1	0.01	−0.05	0.18	−0.11	0.08	0.04	0.02	0.03
CARDS						1	−0.08	0.00	0.21	0.01	–	0.00	–	−0.01
IL2Ra														
SDR							1	0.40	0.35	0.18	0.43	−0.24	0.33	0.65
Go-DARTS							1	0.24	0.24	0.26	0.41	−0.30	0.25	0.60
CARDS							1	0.28	0.09	0.30	–	−0.26	–	0.51
KIM-1														
SDR								1	0.31	0.22	0.31	−0.18	0.46	0.42
G-DARTS								1	0.05	0.14	0.12	−0.15	0.14	0.22
CARDS								1	0.13	0.11	–	−0.09	–	0.36
MB														
SDR									1	0.20	0.56	−0.29	0.59	0.47
GoDARTS									1	0.20	0.35	−0.20	0.40	0.26
CARDS									1	0.13	–	−0.13	–	0.18
NT-proBNP														
SDR										1	0.37	−0.27	0.49	0.27
GoDARTS										1	0.39	−0.30	0.41	0.30
CARDS										1	–	−0.08	–	0.12
SDMA^b^														
SDR											1	−0.47	0.56	0.61
Go-DARTS											1	−0.43	0.42	0.45
THP														
SDR												1	−0.38	−0.40
GoDARTS												1	−0.40	−0.39
CARDS												1	–	−0.29
TnT^b^														
SDR													1	0.50
Go-DARTS													1	0.28

Data are Pearson correlation coefficients for 12 biomarkers shown to be significantly associated in any of the cohorts as well as two additional biomarkers (myoglobin and ferritin) that appear in the sparse panel of Luminex biomarkers

^a^For adrenomedullin, >90% samples were below the detection threshold in SDR

^b^ADMA, SDMA and TnT were not measured in CARDS

ADM, adrenomedullin; CysC, cystatin C; FGF23, fibroblast growth factor 23; FRTN, ferritin; MB, myoglobin; THP, Tamm–Horsfall urinary glycoprotein; TnT, high-sensitivity troponin T; TNFR1, TNF receptor 1

### Generation of a sparse panel from the original case–cohort dataset

As described above, we learned the best platform-specific sets of biomarkers on the original sample set by using forward selection on a given platform with the selection process set to terminate at a maximum of five biomarkers. When restricted only to the ELISA or Luminex biomarkers, the first five biomarkers selected were B2M, KIM-1, myoglobin, NT-proBNP and ferritin. Using only the mass spectrometry biomarkers, the first five biomarkers selected were SDMA–ADMA ratio, α_1_-antitrypsin 2, C16 acylcarnitine, proline and tryptophan. Using nested cross-validation, these panels improved prediction in the original dataset beyond clinical covariates from an AUROC (95% CI) of 0.706 (0.647, 0.764) to 0.846 (0.803, 0.889) for Luminex biomarkers and to 0.806 (0.757, 0.854) for the mass spectrometry biomarkers.

### Predictive performance of sparse biomarker panels in the three validation cohorts

In the validation sets from SDR, GoDARTS and CARDS, the sparse Luminex panels consistently significantly improved prediction in all sample sets on top of clinical covariates, with most of the increment in prediction obtained with the addition of the first two biomarkers, B2M and KIM-1 (Table 4). As shown in Table 4, a combination of B2M and KIM-1 added to clinical covariates, including baseline eGFR and albuminuria, modestly improved prediction, increasing the area under the curve in the SDR, Go-DARTS and CARDS by 0.079, 0.073 and 0.239, respectively. In GoDARTS, but not SDR or CARDS, additional biomarkers myoglobin and NT-proBNP gave a further increment in prediction. The lower AUROC for the CARDS clinical covariates only model can be explained by the fact that participants were matched for age, sex and baseline eGFR so that these variables cannot contribute to the AUROC. Substituting B2M with cystatin C, with which it is highly correlated, achieved a similar increment in prediction in GoDARTS but not in SDR or CARDS (ESM Table 2). In addition, on top of a more extensive set of clinical covariates, the Luminex panel showed a small increment in prediction (ESM Table 3). However, the sparse mass spectrometry-specific panel did not perform well in either the validation SDR or GoDARTS in which it was measured. Comparison of performance with the larger multiplatform panels derived in [6] is reported in ESM Table 4.

**Table 4:** Performance of sparse biomarker panels selected from discovery phase study added sequentially to clinical covariates in the replication cohorts

Biomarker panel	SDR		GoDARTS		CARDS	
	AUROC (95% CI)	Difference in test log_*e*_ likelihood	AUROC (95% CI)	Difference in test log_*e*_ likelihood	AUROC (95% CI)	Difference in test log_*e*_ likelihood
Clinical covariates only	0.628 (0.576, 0.679)	–	0.552 (0.509, 0.595)	–	0.457 (0.371, 0.542)	–
Covariates + B2M	0.690 (0.642, 0.738)	11.6	0.604 (0.563, 0.645)	12.1	0.655 (0.576, 0.735)	11.6
Covariates + B2M + KIM-1	0.707 (0.661, 0.752)	14.2	0.625 (0.584, 0.666)	17.4	0.696 (0.620, 0.773)	12.6
Covariates + B2M + KIM-1+ MB	0.697 (0.651, 0.744)	12.4	0.633 (0.593, 0.673)	19.2	0.695 (0.619, 0.772)	11.9
Covariates + B2M + KIM-1+ MB + NT-proBNP	0.691 (0.643, 0.738)	10.6	0.644 (0.605, 0.682)	21.9	0.690 (0.613, 0.767)	10.6
Covariates + B2M + KIM-1+ MB + NT-proBNP + ferritin	0.689 (0.641, 0.737)	10.5	0.644 (0.606, 0.681)	21.2	0.684 (0.607, 0.762)	10.0
Covariates + top five mass spectrometry biomarkers (SDMA–ADMA ratio, α_1_-antitrypsin 2, C16 acylcarnitine, proline and tryptophan)^a^	0.623 (0.573, 0.672)	<0	0.554 (0.511, 0.596)	<0	–	–

Differences in test log-likelihood are expressed in natural logarithm (log_*e*_) units with respect to clinical covariates only model

Clinical covariates included age, sex, baseline eGFR, albuminuria, HbA_1c_ and calendar time. CARDS models also include a term for treatment allocation

^a^None of the incremental subsets of this panel produced an improvement in predictive performance

MB, myoglobin

**Fig. 2:**
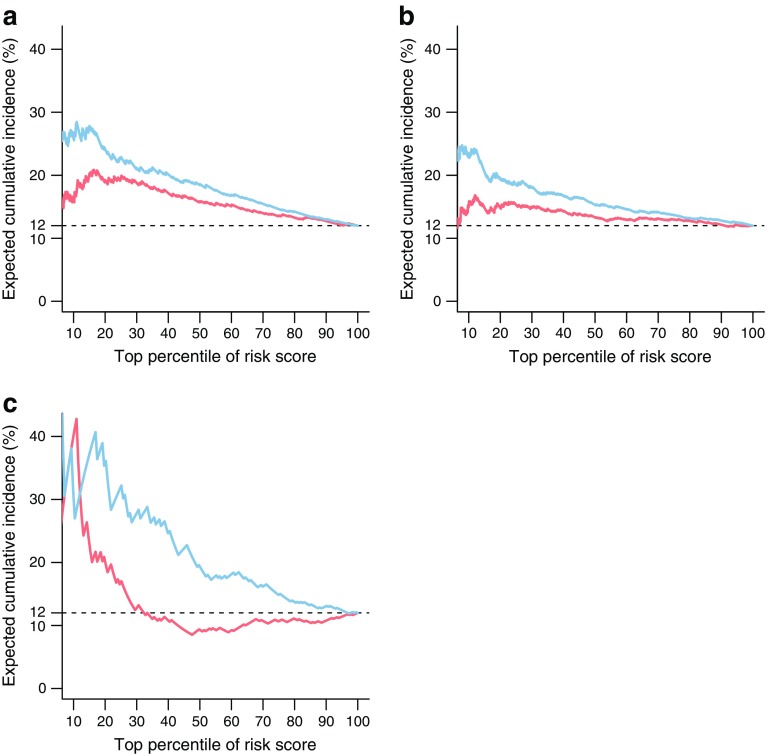
Expected cumulative incidence from the observed 12% (horizontal dashed line) if a trial subsampled the top percentile of possible study entrants according to their risk score for a model containing only clinical covariates (red lines) or a model augmented with B2M and KIM-1 (blue lines), for SDR (**a**), Go-DARTS (**b**) and CARDS (**c**). Clinical covariates are age, sex, baseline eGFR, albuminuria, HbA_1c_ and calendar time. CARDS models also include a term for treatment allocation

The increments in AUROC here are modest. To consider their utility, the role of biomarkers in selection of individuals for entry into a clinical trial can be shown by looking at the predicted event rate enrichment plots (Fig. 2a–c). These plots display the positive predicted value (y-axis) achieved over the percentile of patients sorted by predicted risk score (x-axis). Without any risk stratification, the expected cumulative incidence of a progression event was set to 12%, consistent with what was done in [6]. However, by looking at a subset of individuals with the largest risk scores, a model that included B2M and KIM-1 could yield enrichment for events that would be useful in the context of selection of individuals to be invited into clinical trials. For example, looking at the GoDARTS results (Fig. 2b), selecting people in the top 10% for the biomarkers would enrich the expected event rate from 12% to about 24% (i.e. a doubling in the expected event rate). Across the range of percentiles of risk score, enrichment can be seen for all three studies—the small sample size at the most extreme percentile of 10% showing overlapping lines for CARDS due to small sample size in that part of the range.

## Discussion

We have shown that it is possible to significantly improve prediction of eGFR decline using just two biomarkers—B2M and KIM-1—in combination and that the prediction achieved is similar to that seen in our test cohorts with the previously described larger biomarker panels selected using a discovery cohort [6]. B2M was strongly correlated with a number of other biomarkers, including cystatin C, but substitution with cystatin C did not in general produce the same performance. On the other hand, KIM-1 was not strongly correlated with any of the clinical covariates or other biomarkers we measured.

A range of potentially useful biomarkers of renal disease progression in diabetes have been identified in serum, plasma [2–4], [15] and urine [16, 17] but many studies tested only a small number of biomarkers and few have explored biomarker combinations. Given the pathophysiological complexity of diabetic kidney disease, it is unlikely that one single biomarker can predict its progression [18]. Very few have explored consistency of prediction across cohorts with varying characteristics or under varying sample handling conditions. Here, we have combined the measurement of a wide range of biomarkers in samples from three distinct studies to identify the biomarkers that improve prediction of declining eGFR and thus may be best suited as biomarkers for general use. We have also used logistical considerations, such as keeping the required number of biomarkers low and the desirability for all biomarkers to exist on a single platform, to limit our biomarker selections. This provides a combination of biomarkers that, while it might not maximise prediction in any sample set, significantly improves prediction in all sample sets. Our aim was to identify a non-redundant robust panel of markers that may be of practical relevance, rather than to identify all possible markers associated with progression.

Both B2M and KIM-1 have been widely studied as potential renal biomarkers, though not considered in combination. B2M is fully filtered at the glomerulus and then almost completely reabsorbed in the proximal tubule. In healthy conditions its production is constant, thus making it suitable as a surrogate for eGFR [19, 20]. However, B2M serum levels are elevated in inflammatory conditions, limiting its use as a surrogate [21–23]. There are, however, many reports identifying its potential role as a biomarker for both diabetic kidney disease [24] and end-stage renal disease [25, 26] as well as for CVD [24, 25] and mortality [25, 26]. KIM-1 is also a membrane protein, expressed on the apical membrane of kidney proximal tubule cells. It is a urinary marker of kidney injury and circulating KIM-1 is raised in patients with acute kidney injury [4]. Urinary KIM-1 has shown mixed results as a prognostic biomarker in diabetic kidney disease [27, 28] but glomerular KIM-1 expression is increased in animal models of diabetes [28], associated with elevated plasma levels [29]. Serum KIM-1 also predicts eGFR decline and incidence of end-stage renal disease in type 1 diabetes [4] and is associated with microalbuminuria in type 1 diabetes, suggesting that it may have a role in identifying individuals at risk in early stages of renal disease [30]. B2M is principally a biomarker of filtration while KIM-1 is not, possibly explaining why they work well together in combination. NT-proBNP was also found to add some benefit in the CARDS and GoDARTS cohorts when added to B2M and KIM-1. NT-proBNP is a biomarker for heart failure [31] and may also be a good biomarker for CVD outcomes [32, 33]. However, it is also cleared renally and levels rise as renal function declines [34]. In addition, as noted here, NT-proBNP is not robust to variation in sample handling conditions.

The current study has a number of strengths. By including samples from three studies we identified biomarkers that performed well across populations and studies. By measuring many biomarkers simultaneously, we were able to identify those biomarkers that potentially provide the same information (i.e. B2M and cystatin C) by considering the correlation matrix as well as those that seem to provide additional novel information, such as KIM-1.

Our study illustrates that even when cross-validation is used to avoid overfitting when finding a predictive panel, as we did in defining the large panels in our previous report, this does not guarantee generalisability to other settings. Furthermore, maximising prediction is not the only goal of biomarker discovery. Our study highlights practical considerations such as limiting the panel to a specific assay method and choosing biomarkers that are robust to the sorts of conditions in which they would really be measured. Furthermore, we note that the choice of prediction metric is a complex issue in biomarker studies. Here, not only have we presented the conventional increment in AUROC but also we have shown how the performance increment applies in the context of trial enrichment. An important point is that even modest increments in prediction, as found here, can nonetheless be very useful for enriching event rates in trials.

Our study has focussed on the prediction of serum creatinine-based eGFR decline. Of course, there has been extensive work evaluating the usefulness of other filtration biomarker-based equations including cystatin C and B2M, and their combination, for improving the accuracy of estimation of the underlying true GFR [35, 36]. The development and use of a biomarker panel-based eGFR has recently been advocated for both clinical and trial use [37]. While there are sound arguments and increasing data to support this, we envisage that it will be some time before this is widely approved and adopted as a trial endpoint. In the meantime our data suggest that B2M along with KIM-1 might at least be used for risk stratification into trials using creatinine-based eGFR as part of the endpoint definition.

The study also has limitations. Since there are differences in entry criteria and definition of caseness between our discovery cohort and the cohort sets studied here, we cannot consider this strictly as a replication study. The original biomarker panels were identified based on their power to predict a ≥40% decline in eGFR over a maximum follow-up of 3.5 years whereas in the current study we look at a decline of ≥20% over a longer follow-up period. Thus, we are applying our biomarkers to a much less severe phenotype than previously. Part of the rationale for this study was to explore the use of biomarkers for less extreme phenotypes. We expected that this might diminish associations between biomarkers and outcome. However, we have confirmed that the biomarkers that predict more severe decline in renal function can also predict less severe decline and may be useful at earlier stages of kidney disease. Since a 20% drop in eGFR will be a noisier outcome measure than a 40% drop, this means that we would have had less power to detect biomarker associations. Nevertheless, it would not increase the level of false associations and our strict cross-validation techniques further protect against overfitting. In the GoDARTS and CARDS sample sets in this study the clinical covariates were poor predictors compared with the original discovery case–control study and SDR cohort. However, despite this, addition of the biomarkers increased the AUROC to a similar degree in the SDR and GoDARTS cohorts. We did not have the mass spectrometry biomarkers available in the CARDS samples.

We have shown that the combination of B2M and KIM-1, measured in serum, in addition to clinical covariates, significantly improves prediction of renal function decline in type 2 diabetes on top of clinical data. Use of a larger multiplatform biomarker panel did not consistently improve prediction further.

## Electronic supplementary material

ESM (PDF 776 kb)

## Data Availability

We do not have governance permissions to share individual-level data on which these analyses were conducted. However, for any bona fide requests to audit the validity of the analyses, the verifiable research pipeline which we operate means researchers can request to view the analyses being run and the resultant tabulations. We are also happy to share summary statistics for those wishing to conduct meta-analyses with other studies.

## Abbreviations

ADMA: Asymmetric dimethylarginine
AUROC: Area under the receiver operator characteristic curve
B2M: β_2_-Microglobulin
CARDS: Collaborative Atorvastatin Diabetes Study
CKD: Chronic kidney disease
CVD: Cardiovascular disease
GoDARTS: Genetics of Diabetes Audit and Research in Tayside
IL2Ra: IL-2 receptor α
KIM-1: Kidney injury molecule 1
NT-proBNP: N-terminal prohormone of brain natriuretic peptide
SDMA: Symmetric dimethylarginine
SDR: Scania Diabetes Registry
SUMMIT: Surrogate Markers for Micro- and Macrovascular Hard Endpoints for Innovative Diabetes Tools
