# Supplementary material for: Serum kidney injury molecule 1 and β2-microglobulin perform as well as larger biomarker panels for prediction of rapid decline in renal function in type 2 diabetes
Source: Diabetologia. 2018 Oct 5;62(1):156–68. doi: 10.1007/s00125-018-4741-9 (PMC6290680; doi:10.1007/s00125-018-4741-9)
Supplement: Supplementary file 1 — (PDF 776 kb) [file 125_2018_4741_MOESM1_ESM.pdf]

Electronic Supplementary Materials

ESM Methods

Laboratory measurements of biomarkers

Details of LC-MSMS procedure

At the Mass Spectrometry laboratory lab two protocols are used to yield quantitative and semi-quantitative information on 146 metabolites and peptides and proteins. The WellChild laboratory platform was originally developed to target biomarkers for inherited metabolic disease diagnosis in childhood and this is reflected in some of the biomarkers available on the panel, such as the amino acids and alpha-1-antitrypsin. The platform was built from a series of individual diagnostic clinical assays for class compounds, e.g. acylcarnitines, or single analytes, e,g, creatine. Consequently the individual metabolite assays have been rigorously standardised and subject to internal and external quality control. Stable isotope internal standards for each analyte are included in every sample. This not only allows for any losses during sample preparation but also corrects for any ion suppression and changes in MSMS sensitivity. The tryptic peptide analytical platform is less formally characterised but highly targeted to the more abundant plasma proteins; a single stable isotope peptide is included in every sample. The analytical system for both metabolites and peptides is based on short isocratic chromatography (for positive ion and, metabolites only, negative ion acquisition) and multiple reaction monitoring (MRM) acquisition MSMS. Quantitation is based on the isotope ratio for each analyte in the sample compared to the isotope ratio in aqueous standards run at the beginning and end of each analytical batch assay. The method has been validated against known standards for the entities reported as being quantified (other signals are reported but for as yet unidentified and unvalidated entities some of which show clear associations with eGFR). Some of the quality control metrics are given in table 3. As described above the data from the platform yielded quantification of several biomarkers that were selected for regression procedures as being the most informative for predicting renal disease progression.

For high sensitivity serum metabolite estimation 30µL of aqueous standards, controls and samples are pipetted into 1.8mL polypropylene snap-top Eppendorf tubes. To each tube, 75µL of methanol/water stable isotope mixture 1 followed by 75µL of pure methanol will be added, the tubes capped, vortex mixed for 2-5seconds, and centrifuged at 21,000g at 4°C for 6min. Supernatants, 120µL, will be transferred to a 96 deep well (2mL) polypropylene sample block, sealed, and placed in the autosampler at 8°C ready for analysis by LC electrospray MSMS on an API5500 under Analyst 1.5.2 control. Sample supernatants (3µL) are injected automatically and chromatography performed on an AstecChirobiotic™ T HPLC column 25cm x 2.1mm, 5µm with a 2cm x 4.0mm, 5µm guard column with an isocratic running solvent (acetonitrile:water, 1:1, with 0.025% formic acid) at a flow rate of 250µl/min. Data is acquired in positive ion MRM mode for 15min. Followed by re-injection and data acquisition in negative ion MRM mode for 9min. For Serum tryptic peptide targeted proteomic analysis, 10µL of plasma controls and samples are pipetted as above. To each tube, 40µl of water, 50µL of diluted stable isotope labelled albumin T6 aqueous internal standard, 10µL of acetonitile and 10µL of 1% formic acid are added and mixed on an orbital shaker at RT for 5min. Then 6µL of 1M NH_4CO_3 is added to each tube and vortex mixed for 5 seconds before addition of 25µL of trypsin, vortex mixing, and incubation at 37°C for 1h. After incubation, 200µL of running buffer (acetonitrile:water, 1:1, with 0.025% formic acid) is added to each tube, vortex mixed for 2-5seconds and centrifuged at 21,000g at 4°C for 5min. The supernatants, 200µL, will be transferred to a 96 deep well (2mL) polypropylene sample block as above. Sample supernatants (5µL) will be injected automatically and chromatography performed on two, in series, AstecChirobiotic™ T HPLC Guard columns 2cm x 4.0mm, 5µm with an isocratic running solvent (acetonitrile:water, 1:1, with 0.025% formic acid) at a flow rate of 320µl/min. Data will be acquired in positive ion MRM mode for 10min. Data will be analysed in Analyst version 1.5.2 and MultiQuant version 2.1.

Details of the Myriad RBM platform

At Myriad RBM (MRBM) Luminex technology performs multiplexed, microsphere-based assays in a single reaction vessel by combining optical classification schemes, biochemical assays, flow cytometry and advanced digital signal processing hardware and software. Multiplexing is accomplished by assigning each analyte-specific assay a microsphere set labelled with a unique fluorescence signature. To attain distinct microsphere signatures, two fluorescent dyes, red and far red, are mixed in various combinations using various intensity levels of each dye. Each batch or set of microspheres is encoded with a fluorescent signature by impregnating the microspheres with one of these dye combinations. After the encoding process, an assay-specific capture reagent (i.e., antigens, antibodies, receptors, peptides, enzyme substrates, etc.) is conjugated covalently to each unique set of microspheres. Covalent attachment of the capture reagent to the microspheres is achieved with standard carbodiimide chemistry. After optimizing the parameters of each assay separately, Multi-Analyte Profiles are performed by mixing different sets of the microspheres in a single well of a 96- or 384-format microtiter plate. A small sample volume is added to the well and allowed to react with the microspheres. The assay-specific capture reagent on each individual microsphere binds the analyte of interest. A cocktail of assay-specific, biotinylated detecting reagents (e.g., antigens, antibodies, ligands, etc.), is reacted with the microsphere mixture, followed by a streptavidin-labelled fluorescent "reporter" molecule (typically phycoerythrin). Because the microspheres are in suspension, the assay kinetics are near solution-phase. Finally, the multiplex is washed to remove unbound detecting reagents. After washing, the mixture of microspheres is analyzed using the Luminex 100/200™ instrument. Similar to a flow cytometer, the instrument uses hydrodynamic focusing to pass the microspheres in single file through two laser beams. As each individual microsphere passes through the excitation beams, it is analyzed for size, encoded fluorescence signature and the amount of fluorescence generated in proportion to the analyte. The resulting data stream is interpreted using proprietary data analysis software developed at MRBM. Assays are run in high density multiplexed panels and the Least Detectable Dose (LDD) is determined as the mean +3 standard deviations of 20 blank readings. The LLOQ is determined by the concentration of an analyte where the measurement of analyte demonstrates a coefficient of variation (CV) of 30%. It represents the lowest concentration of analyte that can be measured with a precision better than or equal to 30%. Appropriate dilutions are made to ensure a quantitative measurement within the limits of the assay. An eight (n=8) point standard curve (S1 – S8) is used to obtain quantitative measurements for each sample. Quality Controls (QC's) are run in duplicate along different points of the curve to ensure both accuracy and precision for each analyte.

External QC measures

We undertook a limited study to capture external QC measures on our SUMMIT sample retrieval, transfer, assay and data handling processes for renal biomarkers. This included up to 13 pairs of blinded duplicate samples from the Go-DARTS and SDR sample sets run at the two principle laboratories included in this study. For this data we calculated the intra-class correlation coefficients (ICC) for all biomarkers except Troponin T. We used three levels of ICC to categorise biomarker assays -ICC- ≥0.75 (good), ICC 0.4-0.74 (acceptable) and ICC <0.4 (poor). Of the 42 biomarkers included in the study 39 (93%) were either acceptable or good, 2 performed poorly (1 Luminex and 1 mass spectroscopy biomarkers) and one was not assessed (see table 1 for details).

Biomarker data cleaning and imputation

Data was imputed using a sparse iterative regression approach, where each missing measurement is repeatedly predicted from the observed values using L1-regularised linear and generalised linear models. Our imputation model doesn’t make use of rapid progression status (the target variable to be predicted), in the reconstruction of the missing values. We used a flat prior for imputing the values missing at random and bounded Pareto priors for imputing continuous censored values. When available, we made use of the information concerning detection thresholds in imputing plausible values for censored entries; otherwise these were inferred from the observed values. The iterative imputation model was run 10 times, with initial values of the missing at random entries set by sampling from the marginal distribution of the observed values for each variable. Clinical covariates were imputed first. For biomarkers, the imputation model used all variables including imputed clinical covariates as well as

information concerning the missingness type and the lower and upper detection limits provided by the biomarker laboratories. The dataset used in the analysis was the average of the 10 imputed sets.

Predictive performance of sparse biomarker panels in the three validation cohorts

In addition to calculating the AUROC for each model we also considered difference in test log likelihoods to evaluate the strength of evidence favouring one model over another, based on the asymptotic equivalence of model selection by cross-validation and Akaike Information Criteria (AIC) [1]. For a p-value of 0.05, in a comparison of a two nested models differing by one extra parameter, the difference in the training deviance is distributed according to chi-square distribution with one degree of freedom, and approximately equal to 4. The difference in AIC (the difference in training deviances minus twice the difference in the number of parameters), would be 2 natural log units, and the corresponding approximate difference in the test log-likelihoods will be 1 natural log unit. Therefore, classical statisticians using the likelihood-ratio test to compare training likelihoods of the two models at the significance level of 0.05, should regard a difference of 1 in test log-likelihoods as significant. The more stringent thresholds suggested for Bayes Factors use the cut-offs of 1.2 and 2.3 natural log units as “substantial” and “strong” evidence in favour of the higher-likelihood model [2]. The use of AIC rather than the training likelihoods for model selection has the advantage of penalizing complex models (which helps to prevent overfitting), while the use of the test likelihoods has the additional advantage of not needing to explicitly evaluate the model complexity.

In the SDR and new Go-DARTS datasets we evaluated the performance increment achieved with the larger multiplatform biomarker panels identified previously using the original case-control dataset [3]. The addition of the 14 or 35 biomarker panels to a model including the limited clinical covariates improved the AUROC in both cohorts, with little difference between the two panels (ESM Table 4). Overall, for SDR the best performing panel was the 35 biomarker panel. However, for Go-DARTS the best Luminex-based sparse model out-performed either of these two larger panels when assessed on top of clinical covariates. It is worth noticing that a model based only on the extended set of clinical covariates, including weighted average of past eGFR values, also improved prediction to a similar degree as the biomarker panels added to a basic set of covariates.

References

1. Stone M. An Asymptotic Equivalence of Choice of Model by Cross-Validation and Akaike's Criterion. *Journal of the Royal Statistical Society Series B (Methodological)*. 1977;39:44-7.
2. Jeffreys. The Theory of Probability, 3rd Edition, Oxford 1961.
3. Looker HC, Colombo M, Hess S, et al (2015) Biomarkers of rapid chronic kidney disease progression in type 2 diabetes. Kidney Int 88:888–896.

ESM Tables

ESM Table 1: Distribution of 42 biomarkers analysed by cohort

			SDR					Go-DARTS					CARDS				
Biomarker	Method	Units	Median	IQR		Below detect.	Above detect.	Median	IQR		Below detect.	Above detect.	Median	IQR		Below detect.	Above detect.
Troponin T (high sensitivity) [a]	ELISA	pg/ml	12.3	6.8	24.8	11.8%	0.0%	13.7	8.0	22.9	8.1%	0.0%					
Adrenomedullin	Luminex	ng/ml	-	-	-	92.7%	0.0%	3	2.2	4.0	0.0%	0.0%	0.4	0.4	0.8	61.2%	0.0%
Apolipoprotein D [b]	Luminex	µg/ml	151	117	193.5	0.0%	0.0%	119	91.25	151.75	0.0%	0.0%	120.0	90.5	158.5	0.0%	0.0%
Beta 2 microglobulin	Luminex	µg/ml	3.8	3	4.95	0.0%	0.0%	3.6	2.9	4.7	0.0%	0.0%	2.2	1.9	2.8	0.0%	0.0%
Cystatin C	Luminex	ng/ml	1640	1350	1990	0.0%	0.0%	1620	1340	1969	0.0%	0.0%	1100	940	1355	0.0%	0.0%
Fatty acid binding protein, heart	Luminex	ng/ml	15	8.2	25	12.3%	0.0%	17	11	26	7.0%	0.0%	-	-	-	90.7%	0.0%
Ferritin	Luminex	ng/ml	218.5	123	394.2	0.0%	0.0%	110	53	226	0.0%	0.0%	152.0	78.0	289.0	0.0%	0.0%
Fibroblast Growth Factor 21	Luminex	ng/ml	0.46	0.3	0.725	0.0%	0.0%	0.46	0.28	0.77	0.0%	0.0%	0.4	0.3	0.5	0.0%	0.0%
Fibroblast Growth Factor 23	Luminex	ng/ml	0.04	0.04	0.07	50.7%	0.0%	0.17	0.12	0.27	4.0%	0.0%	0.07	0.07	0.12	54.1%	0.0%
Interleukin 2 Receptor alpha	Luminex	pg/ml	3390	2700	4250	0.0%	0.0%	3600	2852	4558	0.0%	0.0%	2610	2220	3425	0.0%	0.0%
Kidney Injury Molecule 1	Luminex	ng/ml	0.09	0.06	0.17	7.0%	0.0%	0.09	0.06	0.15	9.1%	0.0%	0.08	0.07	0.13	49.7%	0.0%
Latency associated peptide of transforming growth factor beta 1	Luminex	ng/ml	12	9.3	14	0.0%	0.0%	11	9	13	0.0%	0.0%	11.0	8.5	13.0	0.0%	0.0%

Monokine Induced by gamma interferon	Luminex	pg/ml	1020	732	1535	0.0%	0.0%	2205	1522	3550	0.0%	0.0%	989	609	1470	0.0%	0.0%
Myoglobin	Luminex	ng/ml	47	31	66	0.0%	0.0%	66	45	92	0.0%	0.0%	41.0	29.5	56.5	0.0%	0.0%
Neutrophil Gelatinase Associated Lipocalin	Luminex	ng/ml	113.0	72.0	200.5	0.0%	0.0%	498.5	351.2	713.8	0.0%	0.0%	356.0	213.5	642.0	0.0%	0.5%
NT-Pro BNP	Luminex	pg/ml	213	57	663	6.6%	0.0%	724	315	1880	0.9%	0.0%	33	15	153	36.1%	0.0%
Osteopontin	Luminex	ng/ml	9.9	6.7	15	0.4%	0.0%	20	14.25	29	0.0%	0.0%	8.9	6.0	14.0	1.6%	0.0%
Tamm Horsfall Urinary Glycoprotein	Luminex	µg/ml	0.038	0.027	0.0535	0.0%	0.0%	0.031	0.022	0.044	0.0%	0.0%	0.055	0.042	0.071	0.0%	0.0%
Tissue Inhibitor of Metalloproteinases 1	Luminex	ng/ml	232	198	275	0.0%	0.0%	218.5	189	263.8	0.0%	0.0%	151	136	177	0.0%	0.0%
Tumour Necrosis Factor Receptor 1	Luminex	pg/ml	2940	2055	3895	0.0%	0.0%	3025	2380	3970	0.0%	0.0%	2240	1795	2800	0.0%	0.0%
Von Willebrand factor	Luminex	µg/ml	172	133.5	215.5	0.0%	0.0%	127	95	166	0.0%	0.0%	94	70	117	0.0%	0.0%
Asymmetric Dimethylarginine	MSMS	nmol/l	562	500.2	616.8	0.0%	0.0%	591.6	518	690.5	0.0%	0.0%					
Alpha-1 Antitrypsin (1)	MSMS	*	238.62	205.71	270.69	0.0%	0.0%	233.2	197.6	304.1	0.0%	0.0%					
Alpha-1 Antitrypsin (2)	MSMS	*	126.96	82.14	158.05	3.7%	0.0%	134.75	86.06	180.30	5.7%	0.0%					
C16 acylcarnitine	MSMS	nmol/l	349.3	285	441.7	0.0%	0.0%	323.77	253.29	422.95	0.9%	0.0%					
Creatine	MSMS	µmol/l	32.85	20.83	54.25	0.0%	0.0%	48.67	32.33	69.5	0.0%	0.0%					
Creatinine	MSMS	µmol/l	97.75	82.05	117	0.0%	0.0%	106	81.95	130.5	0.0%	0.0%					
Glutamic acid	MSMS	µmol/l	125	101	152.8	0.0%	0.0%	554	494.5	629.5	0.0%	0.0%					

Glutamine	MSMS	µmol/l	634.6	556	715.1	0.0%	0.0%	4.79	4.41	4.99	0.0%	0.0%	
Haptoglobin beta-chain	MSMS	*	1879.35	1298.82	2611.72	0.0%	0.0%	2499.95	1630.20	3506.06	0.0%	0.0%	
Hydroxyproline	MSMS	µmol/l	4.93	3.74	6.385	0.0%	0.0%	6.97	5.13	9.168	0.0%	0.0%	
Hypoxanthine	MSMS	µmol/l	13.452	9.81	17.63	0.0%	0.0%	13.24	9.04	18.16	0.0%	0.0%	
Leucine-rich alpha-2-glycoprotein	MSMS	*	129.77	107.88	159.13	0.0%	0.0%	186.5	148.6	244.5	0.0%	0.0%	
Lysine	MSMS	µmol/l	193.6	170.4	219.1	0.0%	0.0%	229	207.5	256.7	0.0%	0.0%	
Methylmalonic acid	MSMS	nmol/l	150	90	230	7.0%	0.0%	160	120	220	1.3%	0.0%	
N-acetylaspartate	MSMS	nmol/l	438.4	338.1	558.4	1.6%	0.0%	379.56	281.12	490.15	5.7%	0.0%	
Proline	MSMS	µmol/l	233.2	202.6	273.4	0.0%	0.0%	194.75	169.21	230.26	0.0%	0.0%	
Symmetric Dimethylarginine	MSMS	nmol/l	656.8	556.2	808.6	0.0%	0.0%	698.4	572.1	883	0.0%	0.0%	
SDMA:ADMA ratio	MSMS	-	1.187	0.992	1.428			1.203	0.998	1.445			
Thymine[b]	MSMS	nmol/l	66.15	22.94	87.58	34.9%	0.0%	28.94	24.49	40.09	44.0%	0.0%	
Tryptophan	MSMS	µmol/l	53.2	44.56	61.83	0.0%	0.0%	64.93	55.09	74.76	0.0%	0.0%	
Uracil	MSMS	nmol/l	137.13	99.27	176.91	0.0%	0.0%	137.5	99.28	195.75	0.0%	0.0%	

Where units given as * the measure is semi-quantitative with the value being a ratio of the biomarker to a stable isotope of albumin T6.

Where a biomarker is followed by the suffix (1) or (2), this indicates the peptide produced by tryptic digest to which the measured signal relates.

[a] Intraclass correlation coefficient was not assessed

[b] Intraclass correlation coefficient <0.4 (poor)

ESM Table 2: Cross-validated performance of the sparse Luminex biomarker panel in which beta 2 microglobulin is replaced by cystatin C. The clinical covariates model contains age, sex, baseline eGFR, albuminuria, HbA_{1c}, calendar time (CARDS models also include a term for treatment allocation). Differences in test log-likelihood are expressed in natural log units with respect to the clinical covariates only model.

Model	SDR		Go-DARTS		CARDS	
	AUROC (95% CI)	Difference in test log likelihood	AUROC (95% CI)	Difference in test log likelihood	AUROC (95% CI)	Difference in test log likelihood
Clinical covariates only	0.628 (0.576, 0.679)	-	0.552 (0.509, 0.595)	-	0.457 (0.371, 0.542)	-
Covariates + cystatin C	0.666 (0.616, 0.715)	8.7	0.610 (0.569, 0.650)	11.9	0.577 (0.493, 0.660)	3.3
Covariates + cystatin C + KIM-1	0.690 (0.642, 0.737)	12.4	0.630 (0.590, 0.671)	17.8	0.651 (0.570, 0.733)	5.7
Covariates + cystatin C + KIM-1 + myoglobin	0.681 (0.633, 0.729)	10.4	0.641 (0.601, 0.680)	21.0	0.650 (0.569, 0.732)	4.7
Covariates + cystatin C + KIM-1 + myoglobin + NT-ProBNP	0.677 (0.628, 0.725)	8.6	0.650 (0.612, 0.689)	23.2	0.644 (0.562, 0.726)	4.3
Covariates + cystatin C + KIM-1 + myoglobin + NT-ProBNP + ferritin	0.674 (0.625, 0.724)	8.5	0.652 (0.614, 0.689)	22.9	0.639 (0.557, 0.721)	3.5

ESM Table 3: ***Cross-validated performance of sparse biomarker panels added to extended clinical covariates (*** age, sex, baseline eGFR, albuminuria, HbA1c, calendar time, diabetes duration, systolic and diastolic blood pressure, BMI, weighted average of historic eGFR, insulin therapy and smoking status***). CARDS models also include a term for treatment allocation*** *(atorvastatin or placebo) but removed the weighted average of historic eGFR as it was not available*. ***Differences in test log-likelihood are expressed in natural log units with respect to the clinical covariates only model.***

Model	SDR		Go-DARTS		CARDS	
	**AUROC (95% CI)**	**Difference in test log likelihood**	**AUROC (95% CI)**	**Difference in test log likelihood**	**AUROC (95% CI)**	**Difference in test log likelihood**
Extended clinical covariates only	0.693 (0.644, 0.742)	-	0.622 (0.582, 0.661)	-	0.493 (0.408, 0.577)	-
Covariates + B2M	0.712 (0.665, 0.760)	5.4	0.644 (0.605, 0.682)	7.5	0.640 (0.560, 0.720)	9.9
Covariates + B2M + KIM-1	0.733 (0.690, 0.776)	8.1	0.654 (0.615, 0.693)	11.6	0.666 (0.588, 0.745)	9.4
Covariates + B2M + KIM-1 + myoglobin	0.722 (0.678, 0.766)	4.8	0.667 (0.629, 0.704)	15.8	0.667 (0.588, 0.746)	7.9
Covariates + B2M + KIM-1 + myoglobin + NT-ProBNP	0.718 (0.673, 0.764)	3.6	0.668 (0.631, 0.705)	16.7	0.664 (0.585, 0.743)	6.5
Covariates + B2M + KIM-1 + myoglobin + NT-ProBNP + ferritin	0.718 (0.672, 0.763)	3.1	0.668 (0.631, 0.705)	16.1	0.654 (0.575, 0.734)	5.9
Covariates + sparse MSMS biomarker panel [a]	0.680 (0.631, 0.730)	< 0	0.617 (0.578, 0.655)	< 0	*Not measured*	

[a] SDMA:ADMA ratio, alpha-1 antitrypsin (2), C16 acylcarnitine, proline and tryptophan.

ESM Table 4: ***Cross-validated performance of 14 and 35 biomarker panels in models adjusted for clinical covariates (Model 1) and extended clinical covariates (Model 2). Differences in test log likelihood are in natural log units with respect to Model 1.***

	SDR		Go-DARTS	
	**AUROC (95% CI)**	**Difference in test log likelihood**	**AUROC (95% CI)**	**Difference in test log likelihood**
**Model 1 (clinical covariates only)**	0.628 (0.576, 0.679)	-	0.552 (0.509, 0.595)	-
**Model 1 + 14 biomarker panel**	0.702 (0.656, 0.748)	13.7	0.627 (0.587, 0.667)	17.0
**Model 1 + 35 biomarker panel**	0.725 (0.681, 0.769)	18.1	0.619 (0.578, 0.660)	8.0
**Model 2 (extended clinical covariates only)**	0.693 (0.644, 0.742)	13.7	0.622 (0.582, 0.661)	12.9
**Model 2 + 14 biomarker panel**	0.728 (0.683, 0.773)	20.9	0.649 (0.611, 0.687)	19.8
**Model 2 + 35 biomarker panel**	0.734 (0.689, 0.780)	19.0	0.638 (0.599, 0.677)	9.0

Model 1: Clinical covariates include: age, sex, baseline eGFR, albuminuria, HbA_{1c} and calendar time.

Model 2: Extended clinical covariates include: age, sex, baseline eGFR, albuminuria, HbA1c, calendar time, diabetes duration, systolic and diastolic blood pressure, BMI, weighted average of historic eGFR, insulin therapy and smoking status.

14 biomarker panel includes: adrenomedullin, alpha-1-antitrypsin (2), B2M, C-16 acylcarnitine, creatine, creatinine, fatty acid binding protein heart, fibroblast growth factor 21, hydroxyproline, KIM-1, NT-ProBNP, SDMA, SDMA:ADMA ratio, and uracil.

35 biomarker panel includes: alpha-1-antitrypsin (1), alpha-1-antitrypsin (2), apolipoprotein D, C-16 acylcarnitine, creatine, creatinine, cystatin C, fatty acid binding protein heart, fibroblast growth factor 21, fibroblast growth factor 23, glutamic acid, haptoglobin beta-chain, high sensitivity troponin T, hypoxanthine, hydroxyproline, IL2Ra, KIM-1, latency associated peptide of transforming growth factor beta 1, leucine rich alpha 2 glycoprotein, lysine, methylmalonic acid, monokine induced by gamma interferon, N-acetylaspartate, neutrophil gelatinase associated lipocalin, NT-ProBNP, osteopontin, SDMA, SDMA:ADMA ratio, Tamm-Horsfall urinary glycoprotein, thymine, tissue inhibitor of metalloproteinase 1, tryptophan, tumour necrosis factor receptor 1, uracil and Von Willebrand factor.
